# New perspectives on immunological pathways underlying peanut-induced anaphylaxis

**DOI:** 10.1186/2045-7022-1-S1-S68

**Published:** 2011-08-12

**Authors:** Manel Jordana

**Affiliations:** 1 McMaster University, Department of Pathology and Molecular Medicine, Centre for Gene Therapeutics, Hamilton, ON, Canada

## Background

Food-induced anaphylaxis is often a severe allergic reaction characterized by multi-organ dysfunction and a potentially fatal outcome, and accounts for one third to one-half of anaphylactic reactions treated in emergency departments worldwide. Presently, the role of specific effector cells, immunoglobulins and other effector molecules to food-induced anaphylaxis remains to be fully elucidated.

## Methods

To investigate the relative contribution of immunoglobulin-dependent effector pathways to anaphylactic responses to peanut, wild-type and various mutant mice were sensitized with peanut protein and cholera toxin via oral gavage, once weekly for four weeks. Mice were subjected to different cellular depletion and Fc receptor blocking strategies prior to intraperitoneal challenge with peanut one week following the last sensitization. A number of clinical, physiological and immunological outcomes were evaluated.

## Results

Our data indicate that pathways, other than the classical mast cell-IgE pathway, contribute to the full spectrum of anaphylactic reactions to peanut. We show that, remarkably, the combined deficiency of mast cells and macrophages, but not mast cells and basophils, or single depletion of macrophages or basophils, averted nearly all clinical and physiological signs of anaphylaxis. However, the single deletion of mast cells, basophils or macrophages prevented the most significant clinical outcome, death. Furthermore, our data show that using IgE- and IgG1-deficient mice as well as FcRIII blockade, both IgE and IgG1 signalling are necessary to fully abolish anaphylactic responses to peanut. While mast cell responses occurred via IgE and IgG1, macrophage responses were fully mediated through IgG1.

## Conclusions

Peanut-induced anaphylaxis is a process that involves the concerted action of multiple immune effector pathways, and thus interventions targeting one single pathway (e.g. mast cell/IgE) may not be sufficient to fully prevent anaphylactic responses.

